# Long-Term Evaluation of Water Quality and Quantity in a Residential Integrated Rainwater and Greywater Recycling System with Simultaneous Storage and Treatment

**DOI:** 10.1021/acsomega.5c12823

**Published:** 2026-03-02

**Authors:** Andriane de Melo Rodrigues, Edio Damásio da Silva Júnior, Klebber Teodomiro Martins Formiga

**Affiliations:** † Post-Graduate Environmental Science Program, Federal University of Goiás, Esperança Avenue, Goiânia 74690-900, Brazil; ‡ Goiano Federal Institute, 88, 310, Setor Sul, P.O. Box 50, Goiânia, Goiás 74.085-010, Brazil

## Abstract

The present study assessed the operational performance of an integrated rainwater and greywater recycling system (IRGRS) installed in a single-family residence in the town of Rio Verde, central Brazil. The methodology included evaluating both the quantity and quality of water produced by the system over a 16 month monitoring period, with a specific focus on contrasting climatic conditions (well-defined rainy and dry seasons). Operational adjustments were made to the treatment process to improve system performance. The IRGRS integrates rainwater harvesting and greywater pretreatment via a constructed wetland, continuous aeration, cartridge filtration, and ultraviolet disinfection. Water quality parameters remained within national and international reuse standards, with turbidity consistently below 5 NTU, COD under detection limits (<5 mg·L^–1^), and thermotolerant coliforms absent. The system achieved average potable water savings of 41% (minimum 28% and maximum 51%), ensuring self-sufficiency even through six consecutive months without rainfall. Operational stability was confirmed, with low maintenance requirements and reliable performance of treatment components. The study challenges the conventional recommendation of daily greywater disposal, showing that treated greywater can maintain microbiological quality and expand reuse potential. Integrating rainwater and greywater into a single reservoir reduced infrastructure footprint and enhanced system resilience, offering a sustainable alternative for water conservation in regions with pronounced wet and dry seasons.

## Graphical abstract

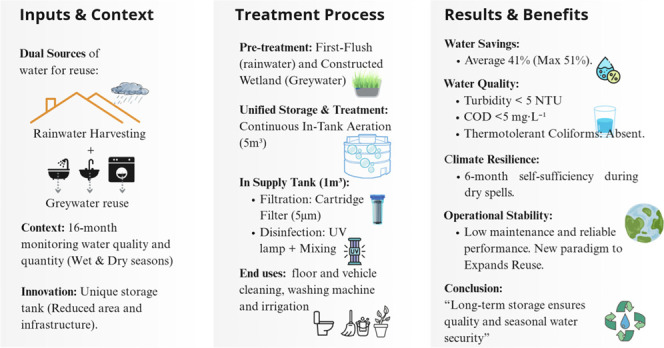

## 1 Introduction

Demographic expansion, accelerated urbanization, and anthropogenic climate change are intensifying pressures on global freshwater systems.^1^ Current estimates indicate that over 2 billion individuals reside in regions experiencing chronic water stress, while approximately 4 billion are subjected to acute scarcity for at least one month annually.^2^ Projections from the IPCC (Intergovernmental Panel on Climate Change)^3^ suggest a marked escalation in the vulnerability of urban water infrastructures by midcentury, particularly under the occurrence of extreme climatic events.

The Central-West Region of Brazil, encompassing the municipality of Rio Verde (southwestern Goiás), exhibits pronounced seasonal contrasts. The wet season (October to April) is characterized by intense and concentrated rainfall, frequently associated with geo-hydrological hazards, whereas the dry season (May to September) is marked by prolonged droughts. Evidence from INPE (National Institute for Space Research)^4^ and CEMADEN (National Center for Monitoring and Alerts of Natural Disasters)^5^ indicates that these climatic extremes have intensified, with increasingly irregular precipitation patterns and droughts of moderate to severe intensity recorded between 2023 and 2024, thereby constraining water availability for both urban consumption and agro-industrial supply.

In this context, unplanned urban expansion and hydrological alterations intensify pressures on water resources, compromising both their quantity and quality.^6,7^ These challenges are further exacerbated by point-source and diffuse pollution,^8–10^ while environmental degradation and climate variability diminish river flows and reduce storage capacities in reservoirs and aquifers, thereby disrupting the equilibrium between supply and demand. As an immediate, yet palliative, response, the drilling of artesian wells in urban areas has been implemented;^11^ however, this practice entails considerable risks, including hydrological imbalance, land subsidence, saline intrusion, and aquifer contamination.^12^ Addressing this scenario requires the diversification of water supply sources through decentralized systems, which can strengthen the sustainability and resilience of existing centralized infrastructures.^13^

Decentralized systems for rainwater harvesting (RWH) and greywater reuse (GWR) are among the most extensively investigated and implemented strategies to address nonpotable water demands.^14–22^ These systems complement conventional water supply networks (WSN), alleviate demand for mains potable water, and contribute to mitigating the impacts of droughts, water scarcity, and flood risks during seasonal rainfall events. Consequently, they constitute decentralized solutions that enhance the resilience and sustainability of urban water supplies in the context of ongoing climate change.^3,23^

Despite advances in the research and application of this technology, critical gaps remain. A systematic review revealed that, among 41 articles, only eight provided detailed assessments of water quality.^24^ Such characterizations are frequently constrained by low sampling frequency (e.g., bimonthly;^25^ 1.2 analyses per month,^26^ short monitoring durations,^27^ and an exclusive reliance on laboratory-scale investigations.^28^ Compilation studies^29–33^ likewise reflect these methodological limitations. In contrast, the present study implemented daily and weekly monitoring over a continuous 16 month period.

Few studies have examined the long-term durability of stored greywater,^34,35^ a critical issue in regions subject to prolonged droughts, when rainwater harvesting becomes unavailable. Although numerous investigations have addressed rainwater or greywater management separately, none of the cited papers have explored the simultaneous storage and integrated treatment of both flows within hybrid reservoirs.^14–18,22,24,26,27,29–33,35–55^ This gap underscores the scarcity of evaluations of systems that integrate rainwater and greywater in a single reservoir with combined treatment, which is essential for assessing efficiency under diverse seasonal contexts—particularly with respect to microbiological control and organoleptic properties, both fundamental for the social acceptability of water reuse.

The literature reports reuse technologies based on membrane separation, such as membrane bioreactors,^17,56–58^ as well as conventional systems employing activated sludge followed by ultrafiltration.^59^ However, these approaches are characterized by high energy requirements, particularly for aeration and membrane cleaning.^60–62^ Although aeration, filtration, and ultraviolet (UV) disinfection are widely documented, especially in the context of wastewater treatment,^63–65^ no study has integrated these processes according to the design concept proposed herein: joint treatment of greywater and rainwater; continuous aeration within the underground reservoir; filtration during pumping to the elevated reservoir using cartridge filters, low-cost and easy to maintain, yet rarely reported in this context;^24^ and disinfection via continuously operating submersible UV lamp, ensuring microbiological safety up to the point of use. This configuration minimizes spatial footprint, preserves water quality, and functions with low energy demand and simplified maintenance, thereby representing an innovative approach to decentralized water reuse.

Beyond water quality, it is essential to assess supply reliability under prolonged drought conditions. This issue is particularly critical in Central Brazil, where the bimodal climate comprises approximately six months of intense rainfall followed by six months of severe drought. Such climatic distinctiveness is seldom addressed in studies of decentralized reuse,^33^ which are typically conducted in regions with milder seasonality.^17,41,66,67^ Hydrological balance analysis, accounting for collected volume, storage dynamics, consumption, and losses, was performed in real time using smart meters. The evaluation confirmed the system’s capacity to fully satisfy the residence’s nonpotable water demand throughout the year, thereby ensuring self-sufficiency during the dry season.

This study employed a long-term (16 month) high-fidelity empirical approach to evaluate the performance of an integrated rainwater and greywater recycling system (IRGRS) implemented in an urban residence in Central Brazil (a region characterized by well-defined wet and dry seasons that remains underrepresented in the literature). This extended evaluation not only strengthens the reliability of the findings but also highlights the resilience of the IRGRS under varying climatic conditions.

The objectives were to assess the quality and quantity of recycled water supplied by the system, the effectiveness of treatment strategies, and the reliability of supply under prolonged drought conditions. The findings contribute to the technical advancement of decentralized water reuse systems and provide evidence to support the formulation of public policies for urban water resource management.

## 2 Methods

### 2.1 Case Study

The study was carried out at a single-family household with two residents, located in Rio Verde, Goiás, Central-West Brazil (17°48′38.11″ S; 50°54′52.45″ W). The property comprises a total impervious area of 311.05 m^2^, including a roofed structure of 203.47 m^2^, and a permeable area of 288.95 m^2^.

The system was monitored between January 2024 and May 2025, covering both the dry (May through September) and rainy seasons (October to April). The region’s climate is markedly seasonal, with rainy (austral) summers and dry winters, as illustrated by the historical series of mean monthly precipitation (mm) and temperature (°C) in the municipality of Rio Verde (Figure 1).

**Figure 1:**
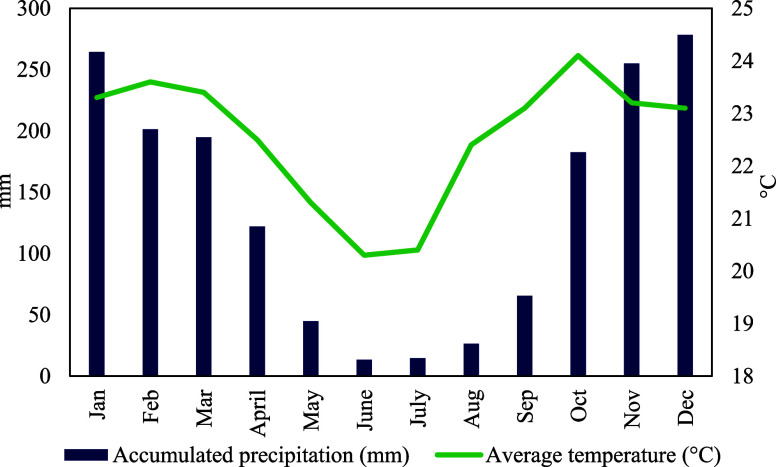
Historical series of mean monthly precipitation (mm) and temperatures (°C) recorded in the municipality of Rio Verde, Goiás (Brazil) between 1961 and 1990. Source: INMET—Instituto Nacional de Meteorologia.^68^

### 2.2 The Integrated Rainwater and Greywater Recycling System: A Case Study and Description

In the IRGRS evaluated in this study (Figure 2), rainwater and greywater are collected separately by gravity flow, directly from their respective sources. Rainwater is harvested from the residential roof, while greywater is generated from showers, washbasins, and the washing machine.

**Figure 2:**
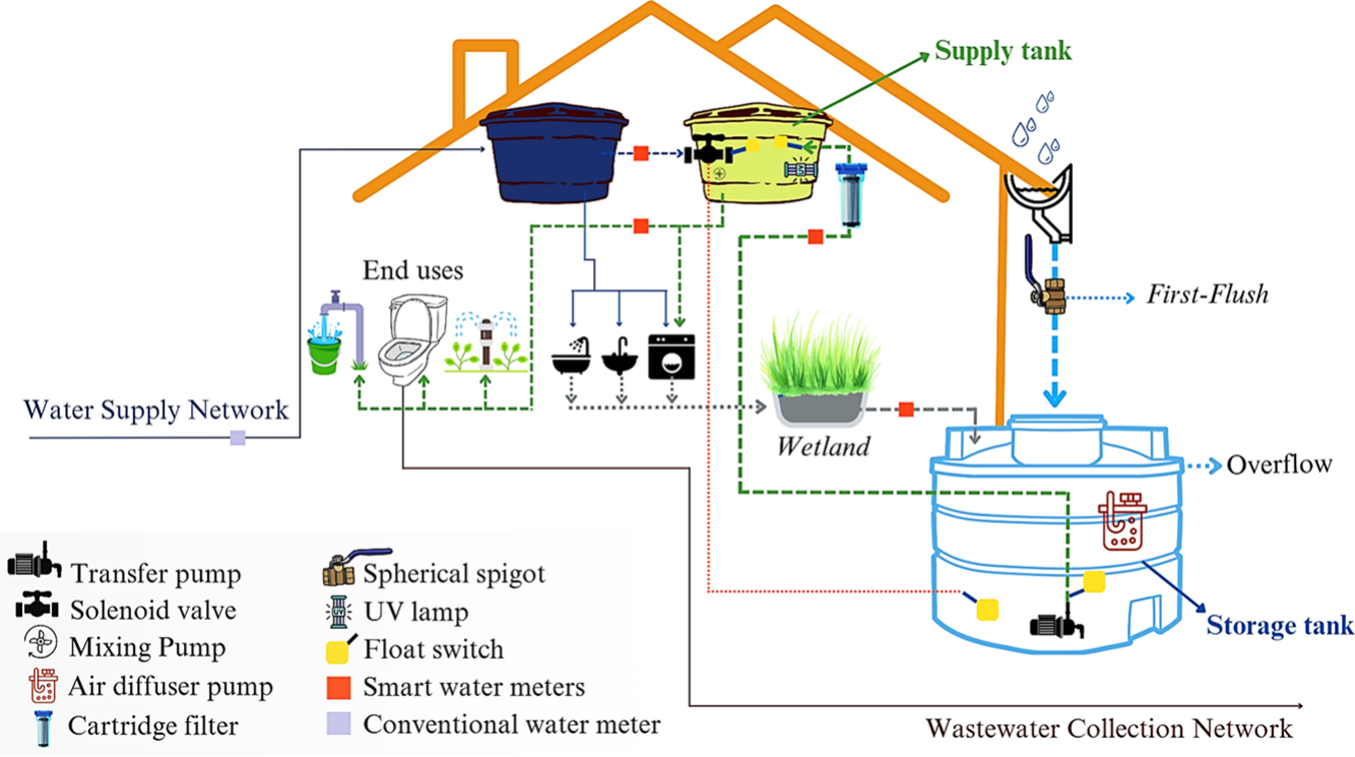
Schematic representation of the IRGRS evaluated in this study. Potable water from the water supply network (WSN) was used for showers, washbasins, kitchen sinks, and washing machines, whereas recycled water was allocated to nonpotable uses, including toilet flushing, floor and vehicle cleaning, washing machines, and irrigation of vegetable plots and gardens.

The initial rainfall (first flush) was discarded to remove atmospheric contaminants and roof debris, serving as a preliminary treatment step prior to storage. This procedure was manually performed using a 100 mm spherical PVC spigot (Fortlev) installed on the rainwater drainage pipe, enabling controlled discharge of the initial runoff. Unlike automatic devices (which systematically discard the first 2 mm of rainwater regardless of surface cleanliness) this manual approach minimizes unnecessary losses by diverting untreated water to the garden rather than the storage tank. The manual first flush was activated only after dry periods exceeding four months, when significant debris accumulation occurred. During the first rainfall, runoff was discharged until the water appeared clear, without color or turbidity, at which point the valve was closed and storage commenced. In the rainy season, frequent precipitation naturally cleaned the roof surface, eliminating the need for repeated flushing.

The greywater, originating from showers, washbasins, and washing machines, was conveyed through 50 mm PVC pipes (Fortlev) to a constructed wetland functioning as an anaerobic biological pretreatment system. The wetland was installed in a 2.3 m^2^ excavated area, sealed with a 1 m^3^ HDPE tank (Fortlev). The filter bed was filled with #1 gravel (a size category of crushed stone, typically 9.5–19 mm in diameter, derived from rocks such as granite, basalt, or gneiss) and planted with the macrophyte *Cyperus proliferus* Lam. (dwarf papyrus). The system was designed to operate with an estimated hydraulic detention time (HDT) of 24 h.

In the following step of the process, the rainwater and greywater are sent to a 5 m^3^ HDPE underground collecting and storage tank (Fortlev), where the two sources were mixed. The size of this tank was validated using the NETUNO 4.0 software.^69^ This tank is aerated continuously by a 45 W submerged pump (Sarlo Better, model SB2700), which injects atmospheric air continuously into the water at a rate of approximately 8.9 L·min^–1^·m^–3^. This process promoted water oxygenation of the water, stimulates the activity of aerobic microorganisms in the degradation of the residual organic matter, and contributes to the mitigation of residual odors, particularly hydrogen sulfide (H_2_S), which are generated by the anaerobic conditions in the constructed wetland treatment unit.

Water from the underground reservoir was pumped to the elevated 1 m^3^ HDPE supply tank (Fortlev) using a submerged 450 W pump (Anauger) with a capacity of 2350 L h^–1^. During this process, sufficient pressure was generated to force the water through a fixed polypropylene (PP) cartridge filter (Fortlev), equipped with replaceable 5 μm pore cartridges, installed in the 32 mm PVC inlet pipes (Fortlev) of the tank. This filtration step removed suspended solids from the recycled water, which was necessary because the aeration process produced a small amount of biomass that had to be eliminated to ensure treatment quality.

An 11 W ultraviolet (UV) lamp (Sarlo Better, Puri Press/G23) was installed underwater in the elevated supply tank for the continuous disinfection of the treated water. An 8 W auxiliary pump (Sarlo Better, SB800A) was also installed in the supply tank to homogenize the water during the disinfection process, to minimize the establishment of dead zones and hydraulic short-circuits. Both these devices operate continuously, 24 h a day, to guarantee the microbiological safety of the water up until its final use. As there was no residual chlorine in the water, continuous disinfection was required, since the recycled water in the residence under study was employed for nonpotable purposes. Specifically, it was used for toilet flushing, floor and vehicle cleaning, washing machines, and irrigation of vegetable plots and gardens.

The IRGR system was designed to pump water from the underground reservoir to the elevated supply tank whenever the water level in the latter reached its minimum threshold. This operation was automated by 15 A polypropylene floating switches (Anauger), which controlled the maximum and minimum levels of both tanks. When the water in the underground reservoir reached its minimum level, the pump’s electric circuit was automatically interrupted.

When the level of recycled water in the underground reservoir was insufficient to meet the residence’s nonpotable demand, a solenoid valve (generic model) was automatically activated by a floating switch, supplying potable water from the WSN to the storage tank, thereby directing water from the public mains to the supply tank.

The aeration and UV disinfection systems were implemented on August 25, 2024, several months after the IRGR system had begun operating, which allowed for the evaluation of the evolution of water quality and the efficiency of these processes over time.

### 2.3 Potable Water Savings

The mean total consumption of water (potable + recycled) by the study residence was 170 L·inhabitant^–1^·day^–1^, and the water saved (reduction in the consumption of potable water from the public WSN) was estimated by eq 1, which provides the percentage of the consumption of potable water substituted by recycled water. This parameter provides a measure of the performance of the IRGR system in terms of the reduction of the dependence of the residence on the public supply.

1 $$Water\;saving\;\left(\%\right)=\frac{V_{reuse}\times 100}{V_{potable}+V_{reuse}}$$

where, water saving (%) = percentage saving in potable consumption from the WSN; *V*_reuse_ = total volume of recycled water consumed by the residence from the supply tank (m^3^), and *V*_potable_ = volume of potable water (in m^3^) consumed by the residence, supplied by the public WSN.

### 2.4 Hydraulic Monitoring

The empirical data on the volume of recycled water produced by the system were collected by hydraulic monitoring with smart water meters installed at different points within the system (Figure 2). Four pulsed output flowmeters (Unijato, wifi capable model SM-WA-HU, IEtecnologia) and an ultrasonic level sensor (wifi capable model SM-WU-HU, IEtecnologia) were used to measure the hydraulic flow and the level of the stored water, respectively. All these devices have integrated dataloggers, which transmit and store data continuously in the cloud on the Monitor IE (IEtecnologia) online platform, with the data being acquired at 1 min intervals.

The volume of the rainwater harvested by the system was measured by an autonomous rain gage (Ciclus WRF-3S, with wifi and Bluetooth connectivity), installed on the roof of the residence. This device records the precipitation instantaneously (mm min^–1^), providing the data necessary to estimate the amount of rainwater harvested. The data was recorded automatically and is available on the weather underground platform.

Smart water meters were used for the hydraulic monitoring of the system, together with an automatic rain gage (Figure 1). These devices allow real-time measurement and remote transmission of water flow data, enhancing the accuracy of system monitoring. Based on the input and output data, the hydraulic dynamics of the system were assessed through the hydrological balance, calculated using eq 1

2 $$V_{rainwater}{+V}_{greywater}{-V}_{reuse}-{\;V}_{ov\mathrm{erf}low}=\frac{dh}{dt}$$

where, *V*_rainwater_ = volume of rainwater entering the storage tank; *V*_greywater_ = volume of pretreated greywater entering the storage tank; *V*_reuse_ = volume of water pumped from the storage tank to the supply tank (equivalent to the amount of recycled water actually consumed); *V*_overflow_ = volume of the water discarded from the storage tank through the overflow mechanism; $\frac{dh}{dt}$ = variation over time in the water level of the storage tank.

### 2.5 Monitoring the Quality of the Water

A total of eight different parameters were measured to determine the quality of the recycled water leaving the supply tank (Table 1), with measurements being taken on either a daily or a weekly basis. The procedures and analytical instruments used to evaluate the quality of the recycled water followed the standard methods for the examination of water and wastewater.^70^ The analyses were run in the Laboratory of Sanitation and the Environment of the Rio Verde campus of the Goiano Federal Institute, Brazil.

**Table 1:** Water Quality Parameters and the Laboratory Procedures Used to Analyze the Samples Collected During the Present Study^a^

parameter	method	equipment	frequency
turbidity	SM 2130 B (nephelometric)	turbidimeter (Akso, TU430)	daily
electrical conductivity	SM 2510 B (conductivity ,meter)	multiparameter apparatus (Akso, AK88)	daily
pH	SM 4500 B (potentiometric)	multiparameter apparatus (Akso, AK88)	daily
temperature	SM 2550 B (direct measurement)	multiparameter apparatus (Akso, AK88)	daily
odor	sensorial assessment	qualitative sensorial analysis (olfactive)	daily
thermotolerant coliforms	SM 9221 B (multiple tubes technique)	laboratory equipment and glassware	weekly
anionic surfactants	Adapted^71^ from SM 5540 C (methylene blue active substances).	spectrophotometry with methylene blue	weekly
chemical oxygen demand (COD)	SM 5220 D (closed reflux, colorimetric)	reactor (Hach, DRB200); spectrophotometer (Kasvi, K37–UV–vis)	weekly

a Analytical methods are referenced according to SM (standard methods for the examination of water and wastewater).^70^

The mean values recorded for the water quality parameters (physicochemical and microbiological variables) were compared between periods using Student’s *t*-test, with a 5% (*p* < 0.05) level of significance. The data were compared between rainy and dry seasons, and between the periods prior to and following the implementation of the aeration and UV disinfection systems. Prior to this analysis, the homogeneity of the variances between groups was assessed using an *F* test, to determine whether *t*-test should be applied for samples with homoscedastic or heteroscedastic variances. All calculations were run in Microsoft Excel.

## 3 Results and Discussion

### 3.1 Hydraulic Performance of the System

The hydraulic performance of the system was evaluated through the hydrological balance of the underground storage tank. Daily, weekly and monthly input of rainwater and greywater was analyzed, along with reuse water (pumped to the elevated supply tank for final use) and losses (due to overflow) (Figure 3).

**Figure 3:**
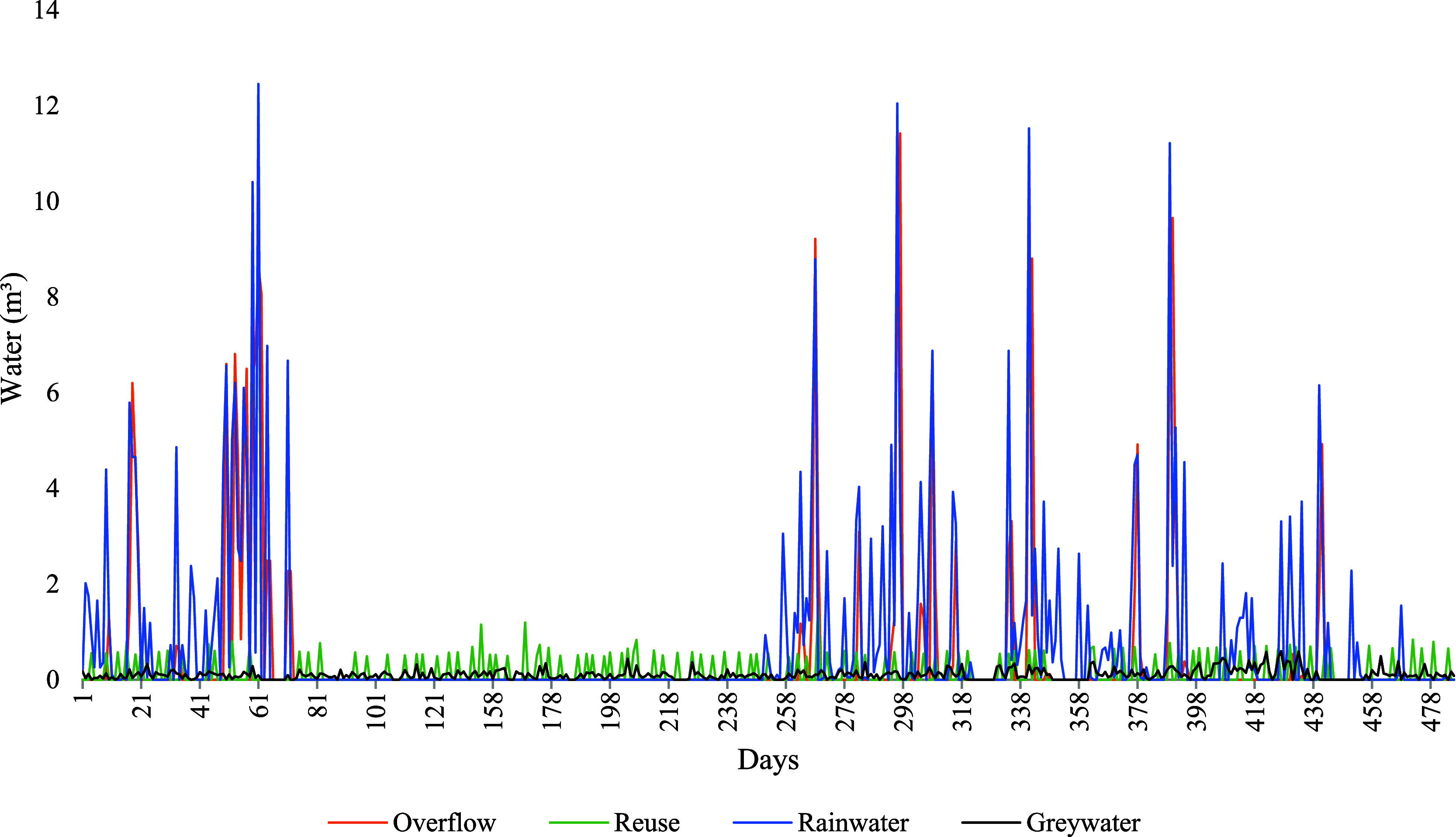
Daily inflows and outflows in the water recycling system installed at the study residence in Rio Verde, Goiás, central Brazil, over the 490 days of the study period (January 2024, through May 2025).

The amount of rainwater harvested varied substantially among the days, and particularly between the well-defined dry and rainy seasons. During the rainy season months, more than 12 m^3^ of rain was harvested on some days, whereas there was no measurable rainfall during much of the dry season. During this latter period, the storage tank depended exclusively on the input of greywater. Given this, overflow events were observed only during the days when rainfall peaked, reflecting the limited storage capacity of the reservoir (approximately 22 mm of accumulated precipitation are sufficient to fill the tank, which has 4.49 m^3^ of effective storage capacity) during intense rains. By contrast, the amount of recycled water used by the residence remained relatively stable over time, reflecting nearly constant demand for nonpotable water.

Seasonal variation between rainy and dry periods is evident (Figure 3). The highest inflows and outflows of water were recorded during the rainy months, from February to March 2024 and October 2024 to early May 2025. On average monthly, 35 m^3^ of rainwater was collected during this period, of which 20 m^3^ was lost through overflow. A low but constant reuse demand (4.1 m^3^ month^–1^) remained unchanged throughout the year. This discrepancy demonstrates that the effective tank capacity of 4.49 m^3^ is sufficient to meet the monthly recycled water consumption of the residence; however, it also indicates that the system was underutilized, since the demand for recycled water could potentially be increased by approximately 8-fold in this season.

The increased demand for recycled water can be strategically directed toward high-consumption uses in buildings, such as the supply and maintenance of swimming pools or artificial lakes. The utilization of excess reservoir water in these structures, in addition to fulfilling landscaping and recreational functions, enhances the operational efficiency of the IRGRS by optimizing storage capacity during periods of extreme precipitation. Allocating surplus recycled water to these auxiliary structures would also prevent discharge into the urban drainage network, thereby contributing directly to flood control.^72^ Furthermore, large-scale mitigation could be achieved through the deployment of multiple similar systems distributed at strategic points within the urban watershed, particularly in areas most vulnerable to flooding.

The system was designed to fulfill a dual function: the reservoir operates as a detention unit, temporarily retaining excess surface runoff and regulating its release to attenuate peak flows and mitigate flood risk, while simultaneously reducing potable water consumption and alleviating pressure on the public supply network, thereby contributing to water scarcity mitigation. The results showed that, even after six consecutive months without rainfall (from April 1 to October 8, 2024), the solenoid valve, installed to activate potable water intake whenever the stored water level dropped below a critical threshold, was never triggered.

The system analyzed in this study, implemented in a single-family residence with only two occupants, exhibited superior performance in average recycled water consumption (68.1 L person^–1^ day^–1^). This result was driven by the smaller population and by the integration of rainwater and greywater treatment and storage within a single reservoir. In comparison, single-family and multifamily buildings operating separate RWHS and GWRS systems showed lower performance: in Portugal,^33^ research reported 41.7 L person^–1^ day^–1^ considering the combined contribution of both RWHS and GWRS, whereas a commercial building in Florianópolis (Brazil) achieved only 9.4 and 7.8 L person^–1^ day^–1^ for RWHS and GWRS, respectively.^54^ Likewise, in Bahnstadt, Germany,^32^ even with 5700 residents, the effective recycled-water use reached only 40 L person^–1^ day^–1^. In multifamily contexts, nonpotable demand is high, and systems are typically segmented by end use (RWHS for laundry and treated GWRS for toilet flushing) thereby reducing complementarity between sources and limiting overall reuse potential compared with fully integrated systems.

The superior efficiency observed in the present study is also associated with the hydrological resilience of the integrated system, which is particularly relevant in strongly seasonal climates. In Rio Verde (Central-West Brazil), the combined use of rainwater and greywater enabled complete self-sufficiency for nonpotable demand over more than six consecutive months without rainfall, supported by the continuous supply of greywater. In Mediterranean climates such as Portugal, where RWHS operation relies heavily on storing winter surpluses to meet summer deficits, shortages were observed during the dry season. In Florianópolis, Brazil, the evenly distributed rainfall throughout the year reduces the attractiveness of greywater recycling, as RWHS alone covers most of the nonpotable demand. In Bahnstadt, Germany, the low annual precipitation (≈715 mm) substantially limited RWHS potential (≈19.25% of nonpotable demand), requiring supplementation with treated greywater, despite low public acceptance (only 20.78% of respondents are willing to install a greywater recycling system, mostly because of public health concerns). These contrasts demonstrate that integrated single-family systems are better positioned to maximize water reuse, whereas multifamily buildings remain strongly constrained by climate conditions, demand profiles, and social acceptance.^32,33,54^

Given the main limitations and operational perspectives discussed, it is reasonable to recommend that the IRGRS at the residential scale be implemented gradually, either through modular reservoir expansion or, alternatively, by directing overflow to swimming pools or ornamental lakes. This strategy provides a practical means of adapting the system to extreme climatic scenarios (e.g., droughts and floods),^24^ while also mitigating the underutilization of recycled water when consumption is significantly lower than the volume captured during rainy periods. From this perspective, the primary priority should be the expansion of recycled water consumption rather than a simple increase in storage capacity.

### 3.2 Savings of Potable Water

The total water consumption of the study residence (including both potable and recycled water), the nonpotable use of recycled water, and the associated savings varied considerably across the different months of the study period (Figure 4), between February 2024, and May 2025. The average total consumption over the 16 month monitoring period was approximately 10.1 m^3^ per month, of which around 4.1 m^3^ were supplied by recycled water and 6.0 m^3^ by potable water. This corresponded to an average reduction of approximately 41% in potable water consumption from WSN usage.

**Figure 4:**
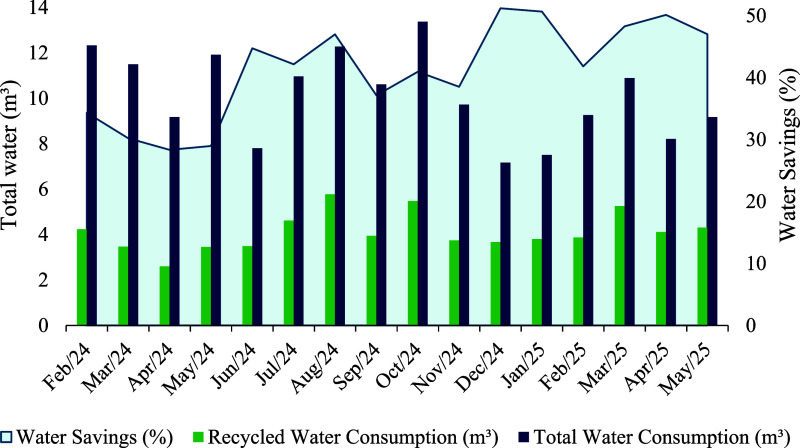
Monthly total water consumption, recycled water consumption, and mean savings in potable water (%) from WSN usage at the study residence in Rio Verde, Goiás, central Brazil, between February 2024 and May 2025.

Between 28% and 51% of potable water consumption from the WSN was saved each month during the study period. The greatest savings were observed in November 2024 and in January and March 2025, due to the increased availability of rainwater. The entry of rainwater into the system improved the quality of the final reuse water, making it possible to use it for laundry.

Previous studies have reported highly variable efficiencies in water reuse systems, reflecting the influence of local context, system scale, and reuse patterns. Integrated RWH–GWR systems achieved savings of 28.3%, whereas RWH alone yielded only 6.9–10.4% from roof areas of 600–900 m^2^, a performance largely attributed to low regional precipitation (774.3 mm annually, with a uniform monthly average of 64.5 mm).^16,21^ At one site, rainwater harvesting satisfied 34.6% of demand, while a standalone greywater system reached only 28%, likely due to insufficient reservoir capacity (250 L), a restriction imposed to prevent nondisinfected effluent (treated in a constructed wetland) from being stored for more than 24 h.^39^ In another study,^73^ inadequate infrastructure, including the same 250 L storage limitation, further reduced greywater system efficiency to just 3.05%.

A particularly relevant case reported a negative balance of −8.5% to −10.0% in integrated systems with hybrid storage tanks (rainwater + greywater), where the potable water required for filter backwashing exceeded the volume recovered by the system implemented in a monitored commercial building.^74^ These contrasts underscore that the efficiency of reuse systems depends critically on supply-demand compatibility, technical design, storage capacity, treatment processes, and consumer profile.

In southern Brazil, the use of nonpotable water for laundry and toilet flushing yields substantial reductions in potable water consumption: 34.6% for RWH and 28.0% for GWR in single-family households;^39^ 43% and 24%, respectively in other study;^42^ and approximately 38% when both systems operate in combination in other reserach.^26^ Although RWHS are particularly efficient, their performance decreases in multifamily buildings due to higher demand, resulting in savings of only 6–7%, depending on demand and storage capacity.^54^ Greywater reuse systems show a similar trend: while they achieve 26–30% savings in single-family households, their contribution in multifamily buildings also declines to around 6%.^39,54^

Overall system performance, however, remains strongly climate dependent. While the regular rainfall distribution in southern Brazil favors rainwater harvesting, regions such as the Brazilian Center-West and Mediterranean climates like Portugal, characterized by prolonged dry seasons and, in some cases, projected reductions in annual precipitation, tend to benefit more from the stable supply provided by GWRS during extended drought periods.^33^

In the present study, recycled water use for nonpotable purposes exhibited clear seasonal variation (Figure 5). Between July and December 2024, most of the recycled water was allocated to irrigation and floor washing, peaking at 3.9 m^3^ in October 2024 during the dry season. From January 2025 onward, consumption shifted toward toilet flushing and laundry, driven by increased rainfall and reduced dust accumulation. In March 2025, 4.3 m^3^ were used for these purposes, representing the highest monthly volume recorded during the study period.

**Figure 5:**
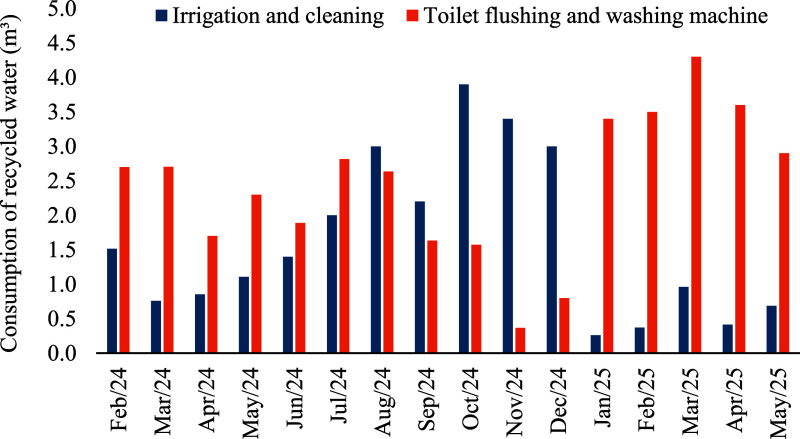
Monthly consumption of recycled water used for nonpotable drinking purposes in the study residence in Rio Verde, central Brazil, between February 2024 and May 2025.

Ultimately, monthly savings of up to 50% in potable water consumption from WSN underscore the effectiveness of the IRGRS in consistently meeting the nonpotable demands of the study residence. These uses include irrigation and cleaning (approximately 40% of recycled water consumption) as well as toilet flushing and washing machine operation (approximately 60%). The continuous supply of recycled water for nonpotable purposes throughout the year further demonstrates the IRGRS’s resilience to prolonged drought events, thereby enhancing its potential for climate-change adaptation. Successful adaptation measures strengthen system resilience while reducing vulnerability to multiple environmental stressors.^75,76^

### 3.3 Assessment of the Quality of the Recycled Water

The quality of recycled water was assessed over a 490 day monitoring period, with daily measurements of turbidity, EC, pH, and temperature, and weekly analyses of TTC, COD, and anionic surfactants. The study compared the rainy season (January 29 to April 14, 2024; October 8, 2024 to May 9, 2025) with the dry season (April 11 to October 7, 2024; May 10 to 31, 2025), focusing on the impact of continuous aeration and UV disinfection implemented on August 25, 2025. The dry season was defined as ≥15 consecutive days with daily rainfall <1 mm and monthly accumulation <30 mm. The integration of aeration and UV technologies was required to address odor problems and increases in turbidity, COD and TTC levels at the onset of the dry season. This deterioration in quality was attributed to the absence of rainwater dilution, resulting in a highly concentrated effluent composed exclusively of greywater. These conditions required the installation of a continuous aeration unit in the storage tank and a UV lamp in the supply tank to ensure adequate treatment and compliance with reuse standards.

Figures 6 and 7 present the statistically significant effects of ultraviolet (UV) radiation and aeration, while also confirming the influence of seasonal climatic variation on turbidity (*p* < 0.001, Student’s *t*-test). Full results of the Student’s *t*-test are provided in the Supporting Information. The “dry–before” condition exhibited the highest variability, with a median turbidity of approximately 18 NTU, attributable to greywater concentration during periods of reduced rainfall. Following the treatment interventions, recycled water achieved median turbidity values of 2.5 NTU in the dry season and 0.3 NTU in the rainy season. Aeration supplies the oxygen required for aerobic microorganisms to metabolize dissolved and suspended organic matter, thereby promoting reductions in turbidity and COD.^77^ These values are consistent with ordinance no. 888/2021 of the Brazilian Ministry of Health,^78^ which stipulates turbidity thresholds of <0.5 NTU for filtered water and <5 NTU for distribution systems. Collectively, these findings highlight the effectiveness of integrated treatment strategies in sustaining water quality standards under contrasting hydrological conditions.

**Figure 6:**
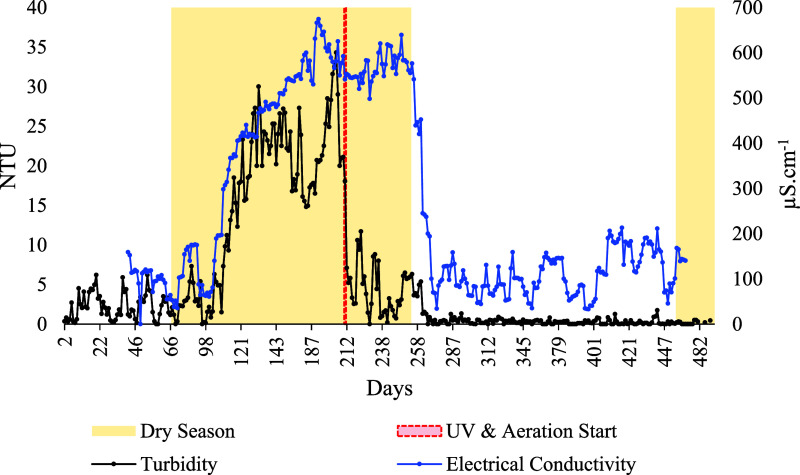
Daily variation in the turbidity (NTU) and electrical conductivity (μS.cm^–1^) recorded over the 490 days of the study period in Rio Verde, Goiás, central Brazil.

**Figure 7:**
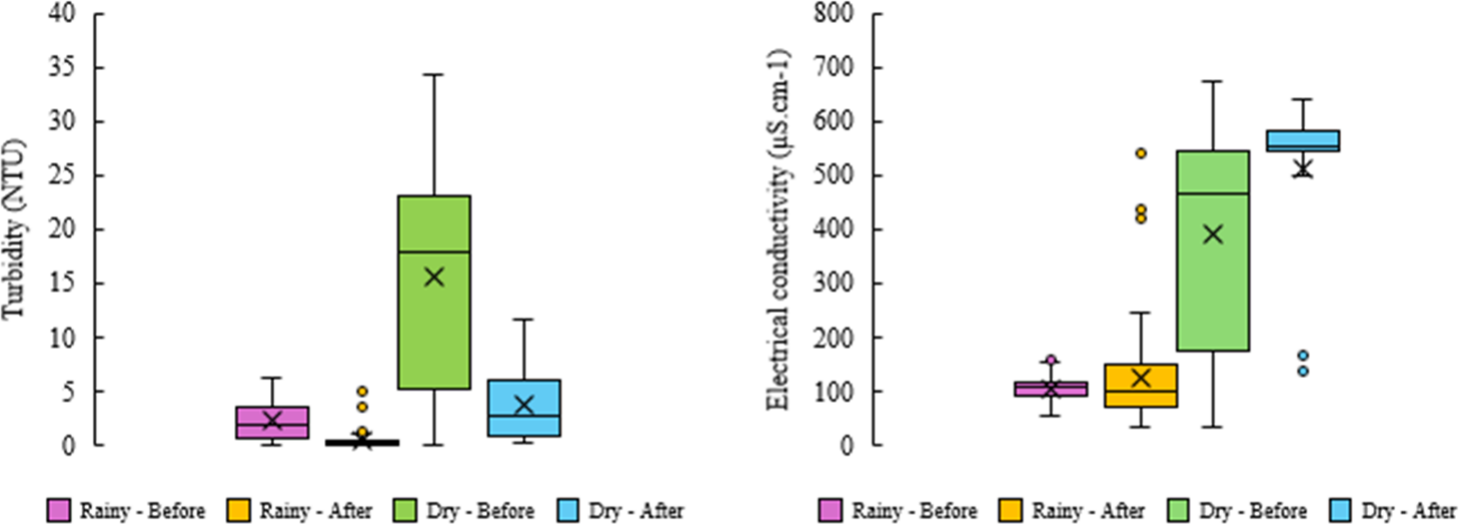
Variation in turbidity (NTU) and electrical conductivity (μS·cm^–1^) during rainy and dry seasons, before and after the implementation of the UV and aeration system.

Literature reports considerable variation in turbidity levels depending on water source and the level of treatment. Values ranging from 0.2 to 1349 NTU for rainwater (RW) and 60 to 240 NTU for greywater (GW) have been documented.^29,79^ Intermediate ranges have also been reported,^20,28^ with turbidity of 1–42 NTU (RW) and 29–185 NTU (GW), as well as 1–24 NTU (RW) and 130–167 NTU (GW). By contrast, studies involving more intensive treatment processes have reported residual turbidity below 2 NTU, reflecting greater stability in water quality.^26,31^

These values also comply with the limits recommended by international guidelines, such as USEPA^80,81^ and ISO 16,075-2,^82^ which generally establish an average turbidity of 2 NTU and a maximum of 5 NTU for agricultural reuse. Furthermore, the results meet the more stringent requirements of British Columbia,^83^ which stipulate turbidity levels below 2 NTU, and fall within the “no risk” range (0–1 NTU) defined by South African guidelines.^84^ In the national context, the recorded turbidity values were substantially lower than the 5 NTU threshold established by Brazilian standards ABNT NBR 15,527 ^85^ and NBR 16,783 ^86^ for nonpotable reuse of rainwater and alternative water sources in residential buildings.

The physicochemical profile, particularly EC, was primarily governed by seasonal dynamics rather than treatment interventions, since UV irradiation and aeration do not target dissolved ion removal. Accordingly, Figures 6 and 7 show that the “dry” phase exhibited the highest variability and mean EC levels, reflecting increased ionic concentrations due to reduced dilution. This seasonal difference was statistically significant (*p* < 0.001, Student’s *t*-test), irrespective of the intervention stage. Notably, the peak EC value of 650 μS·cm^–1^ recorded during the dry season remained well within the Australian guidelines for water recycling (AGWR) range (200–2900 μS·cm^–1^)^87^ and is consistent with previous studies.^28,29^

The parameters pH and temperature remained stable and within regulatory compliance throughout the study (Figures 8 and 9). The pH consistently remained within the neutral range (6–8), consistent with previous research and fully compliant with Brazilian (GM/MS 888/2021)^78^ and USEPA guidelines^80,81^ for both potable and reuse water. Similarly, water temperature remained below 35 °C even during the hottest months (October and November), with fluctuations attributed to seasonal transitions (spring to summer) rather than operational factors. These results are consistent with the limits recommended for potable water in Brazil.^78^

**Figure 8:**
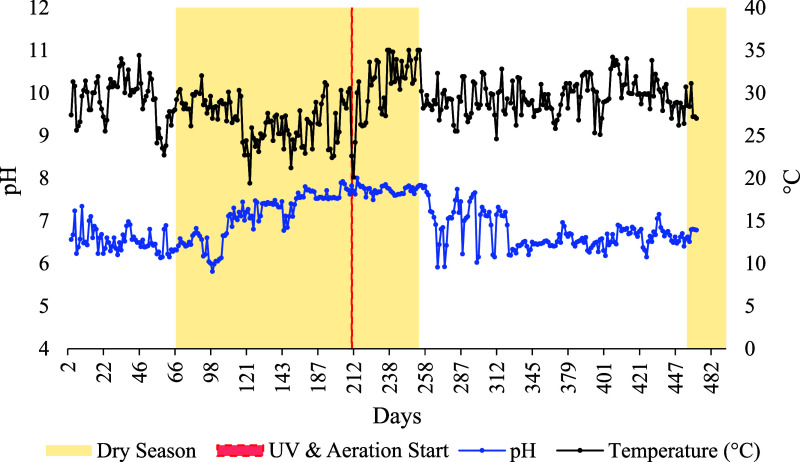
Daily variation in the pH and temperature (C°) recorded over the 490 days of the study period in Rio Verde, Goiás, central Brazil.

**Figure 9:**
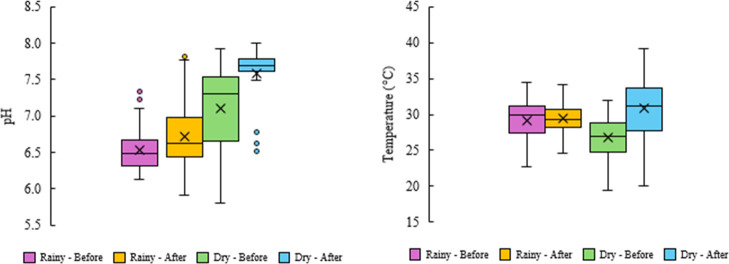
Variation in pH and temperature (°C) across rainy and dry seasons, before and after implementation of the UV and aeration system.

International guidelines generally recommend a neutral to slightly alkaline pH range for reclaimed water. South African and Brazilian standards^84,86^ permit a broad interval (6.0–9.0), while Australian guidelines^87^ extend up to 9.8. By contrast, the USEPA^80,81^ is notably stricter, requiring a narrower range of 6.5–7.5. The consistent neutrality of the pH (6–8) observed in this study agrees with previous research.^28,79^ Regarding temperature, there is consensus among agencies such as the USEPA^80,81^ and South African guidelines^84^ that levels exceeding 30 °C should be avoided, as they promote microbial proliferation in water intended for reuse.

Before the implementation of continuous aeration and UV disinfection, odors were perceptible in the recycled water. This outcome, confirmed by residents’ perception, suggests the effective oxidation of volatile sulfur compounds presumably accumulated during anaerobic pretreatment in the constructed wetland. Notably, the aeration rate applied in the IRGRS in this study (8.9 L·min^–1^·m^–3^) was approximately 14× higher than that reported in another study^88^ (0.49–0.63 L·min^–1^·m^–3^), a difference that likely accounts for the efficient removal of the observed odors.

Such conditions, particularly the neutral pH verified in this system, favor the conversion of sulfur compounds predominantly into elemental sulfur and polysulfides, thereby minimizing sulfate formation.^88^ This mechanism is further supported by findings indicating that microaeration promotes the stable formation of elemental sulfur, mitigating the release of volatile sulfurous compounds.^89^ Consequently, the aeration strategy ensured that the reclaimed water was odorless, in compliance with international guidelines, including those established by South African standards^84^ and the USEPA.^80,81^

COD and anionic surfactant concentrations were monitored weekly over a 70 week period (January 28, 2024 to May 31, 2025), as shown in Figures 10 and 11. Throughout the study, COD levels did not exceed 76.7 mg·L^–1^, aligning with the lower end of values reported in comparable literature (76–675 mg·L^–1^).^29,79^ Following the implementation of UV disinfection and aeration, COD concentrations were consistently reduced to <5 mg·L^–1^, indicating a removal efficiency superior to that observed in most previous studies.^28,29^ This reduction was statistically significant in both dry (*p* < 0.005) and rainy (*p* < 0.001) seasons. Post-treatment median values remained below the method detection limit (<5 mg·L^–1^), irrespective of seasonal variation, and were well within the regulatory threshold established by USEPA (50 mg·L^–1^). Moreover, the results complied with BOD_5,20_ standards defined by ISO (5–10 mg·L^–1^)^82^ and ABNT NBR 16,783 (BOD_5,20_ < 20 mg·L^–1^),^86^ reinforcing the effectiveness of the treatment strategy in meeting both international and national water quality criteria.

**Figure 10:**
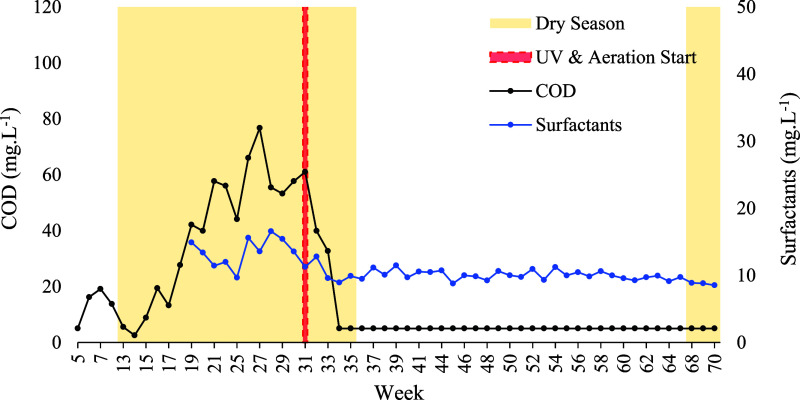
Weekly variation in the COD and concentration of surfactants (mg·L^–1^) recorded over the 70 weeks of the study period in Rio Verde, Goiás, central Brazil.

**Figure 11:**
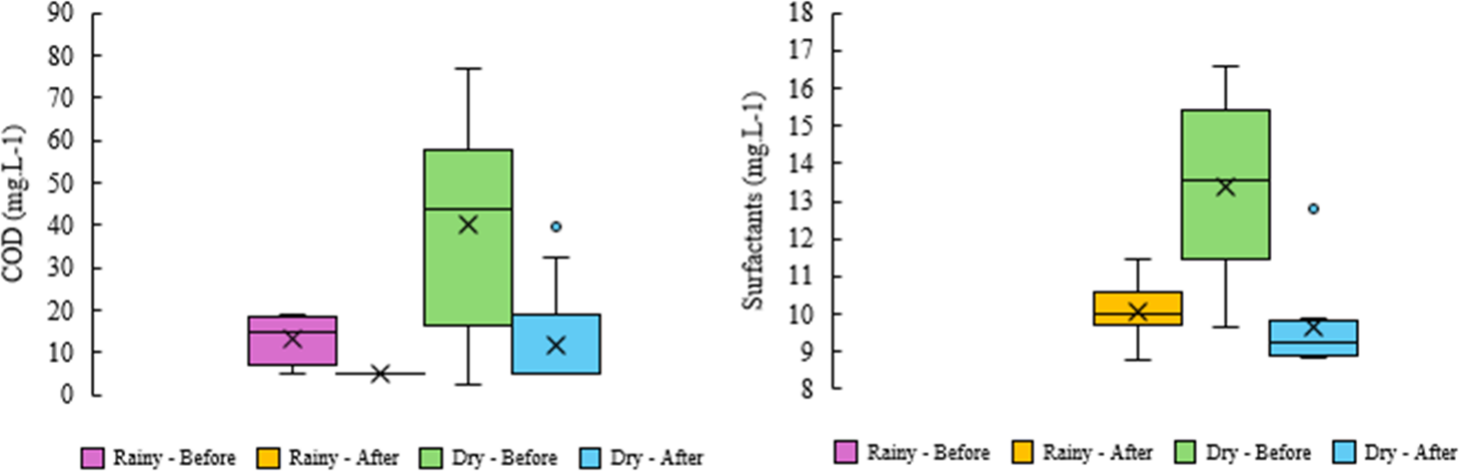
Variation in COD and anionic surfactant concentration (mg·L^–1^) across rainy and dry seasons, before and after implementation of the UV and aeration system.

The implementation of UV disinfection and aeration significantly enhanced anionic surfactant removal, with effectiveness observed during the dry season (*p* < 0.005, Student’s *t*-test). While anionic surfactants generally exhibit higher degradation rates compared to nonionic and cationic surfactants, compounds such as LAS (Linear alkylbenzenesulfonates) can persist under anaerobic conditions. This persistence may explain the measured concentrations of anionic surfactants in the final reuse water, given that part of the biological treatment operated under anaerobic conditions (constructed wetland). Although aeration was applied in the tank, the hydraulic retention time was likely insufficient to achieve complete degradation. Evidence indicates that LAS degrades in sludge-amended soils with a half-life ranging from 7 to 33 days.^90^ Therefore, it is reasonable to assume that the surfactants present in recycled water used for irrigating vegetable and flower gardens may have undergone degradation within approximately one month after contact with the soil.

Post-UV and aeration surfactant concentrations remained at ≈10 mg L^–1^. This level is substantially higher than the most restrictive international guidelines for anionic surfactants, such as those established in Italy^91^ (0.5 mg L^–1^), the AGWR^87^ (0.2 mg L^–1^), and the USEPA Water Reuse Guidelines^80,81^ (<1 mg L^–1^). At present, surfactants are not regulated by Brazilian standards.^85,86^ The elevated concentration highlights the need for complementary treatment when reclaimed water is intended for sensitive reuse applications. Advanced Oxidation Processes (AOPs), such as UV/H_2_O_2_ or O_3_/UV, are recommended for complete degradation, as they generate highly reactive hydroxyl radicals capable of mineralizing these recalcitrant compounds.^92^

The density of thermotolerant coliforms (Figures 12 and 13) peaked at log_10_ 5.08 MPN·100 mL^–1^ during the dry season, prior to the implementation of continuous aeration and ultraviolet disinfection. UV radiation inactivates coliforms by damaging their DNA, preventing replication and ultimately leading to cell death.^93^ These values are consistent with concentrations reported in untreated rainwater (log_10_ 3.70–4.78 CFU·100 mL^–1^) and greywater (up to log_10_ 5.19 CFU·100 mL^–1^).^25,31^ From day 211 onward, following the introduction of aeration and UV disinfection, coliform levels decreased by 4.07 log, stabilizing between 0 and 2 MPN·100 mL^–1^, values close to or below the quantification limit. This reduction was statistically significant (*p* < 0.0001 in the rainy season; *p* < 0.005 in the dry season, Student’s *t*-test) and met World Health Organization (WHO) benchmarks for restricted irrigation (3–4 log reduction) as well as unrestricted irrigation (≤1000 *E. coli*·100 mL^–1^).^94^

**Figure 12:**
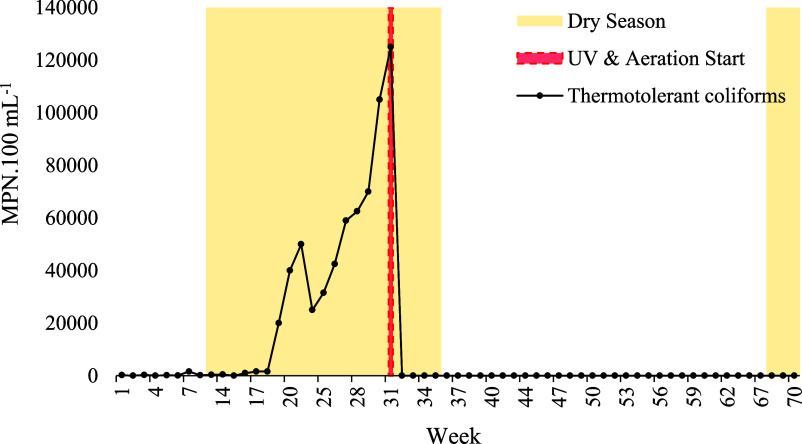
Weekly variation in the thermotolerant coliform concentration (MPN.mL-1) recorded over the 70 weeks of the study period in Rio Verde, Goiás, central Brazil.

**Figure 13:**
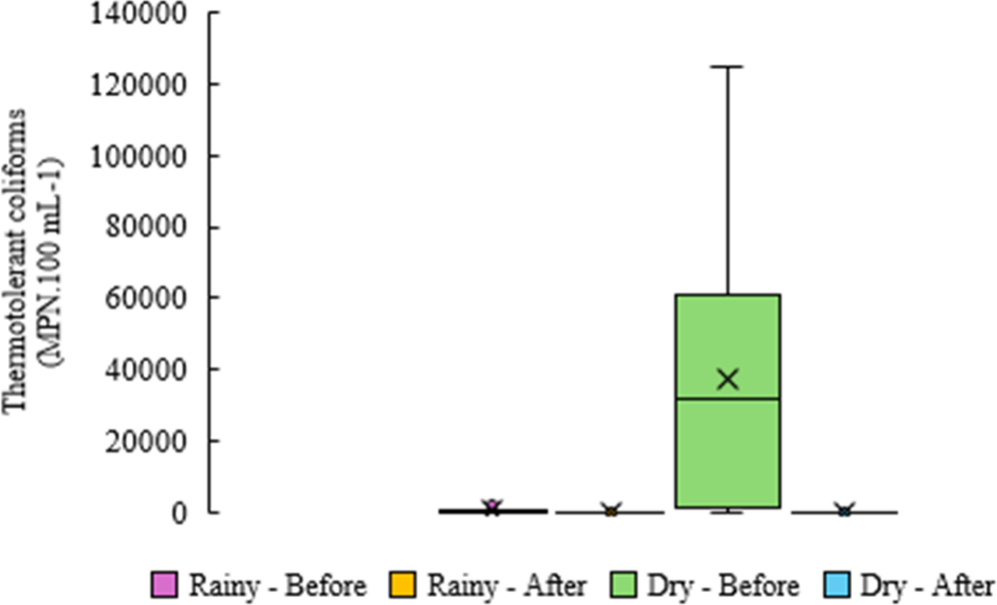
Variation in thermotolerant coliform concentration (MPN·mL^–1^) across rainy and dry seasons, before and after implementation of the UV and aeration system.

Overall, mean concentrations declined from >30,000 MPN·100 mL^–1^ to 7 MPN·100 mL^–1^ post-treatment using UV, fully complying with international reuse standards. These include the USEPA^80,81^ threshold of 2.2 MPN·100 mL^–1^ for unrestricted daily reuse, ISO guidelines^82^ for agricultural irrigation (10–100 MPN·100 mL^–1^), and South African criteria,^84^ which identify sanitary risks above 10 MPN·100 mL^–1^.

Operational norms for water reuse in agriculture and urban applications vary among national and international regulatory bodies (Supporting Information). In comparison, the results of this study demonstrate that the water produced by the IRGR system achieved adequate quality for most reuse purposes.

In Brazil, regulations remain limited and fragmented. The parameters defined by the Brazilian Association for Technical Norms (ABNT), while relevant, lack integration with nationwide public policy. The absence of specific federal legislation contributes to heterogeneity among states, only a few of which have complementary rules for reuse.^95^ The Guidelines for the Reuse of Water in Brazil,^96^ published in 2025 by the Brazilian Institute for Water Reuse, represent an important technical advance by introducing a risk-based approach, with differentiated standards according to use (urban, agricultural, industrial) and monitoring plans proportional to system scale and risk level.

Within this framework, the recycled water analyzed in this study was compatible with the requisites of CONAMA Resolution n° 357/2005 ^97^ meeting the standards of the special class (absence or minimal thermotolerant coliforms) and, during the rainy season, satisfying class 1 criteria (≤200 MPN·100 mL^–1^ in 80% of samples). These classifications permit uses such as recreation, irrigation of raw-consumed vegetables, aquaculture, and fisheries with simple disinfection.

Although technical literature frequently recommends discarding greywater stored for more than 24 h due to the risk of exponential bacterial growth,^34^ a guideline adopted in several subsequent studies,^27,30,35,36,44,48,98,99^ the present study demonstrates that adequate microbiological safety can be achieved through appropriate treatment rather than disposal. The stored volumes of greywater (in the absence of rainfall, when the system received only greywater) were essential to ensure a continuous supply for nonpotable use in the residence during the prolonged dry period.

### 3.4 Operational and Perceptive Features

The maintenance of the supply tank included replacement of the filter cartridge, the UV lamp and cleaning of the supply reservoir. The first intervention was conducted in week 8 (March 25th, 2024) and involved the installation of a filter cartridge with 1 μm pores and the cleaning of the tank, which was motivated by the elevated count of thermotolerant coliforms during the preceding week (Figure 12). Although the microbiological response was the sole parameter explicitly represented to assess the effects of maintenance, other physicochemical indicators were also affected. Notably, reductions in turbidity and COD were observed in the weeks following the intervention.

In week 14, the transfer pump failed, given that the pressure generated by this pump was too weak to overcome the resistance caused by the fine-pored cartridge filter, compromising the hydraulic performance of the system. This problem was resolved by replacing the pump with a more powerful model (450 W), while the filter cartridge was replaced with a refill with 5 μm pores. The concentration of thermotolerant coliforms increased over the subsequent weeks, a scenario aggravated by the drought conditions. The abrupt reduction in the coliform count in week 24 coincided with a 15 day period during which the inhabitants of the study residence were absent, suggesting the possible influence of the temporary stagnation of the water.

A comprehensive program of maintenance was carried out in week 31 (August 25th, 2024), including the installation of the UV lamp and the auxiliary pump, including the cleaning of the storage tank and the replacement of the 5 μm filter cartridge. From this point onward, the quality of the water remained stable until week 69 (May 20th, 2025), when a slight level of contamination (23 MPN.100 mL^–1^) was detected, which signaled the need for new preventive maintenance. It is important to note that all these interventions were implemented based on the monitoring of the quality of the water.

Disinfection with UV lamps, associated with simple pretreatments, is effective for the removal of organic matter, turbidity, and pathogenic microorganisms,^100^ making the water adequate for agricultural reuse. However, this study also documented the formation of solid incrustations on the surface of the installation, which required periodic cleaning to ensure adequate performance. In the present study, while incrustations were observed on the surface of the underwater UV lamp, no loss of water quality was detected. It seems likely that the impact of these incrustations was minimized due to the high theoretical dose of UV radiation (250–335 mJ·cm^–2^, UV–C) accumulated over the 24 h of continuous exposure. This estimate is based on the use of an 11 W lamp (3.3 W of UV–C at 254 nm), submerged in up to 850 L of water, with slow agitation provided by an 8 W underwater pump.^101^

The ultraviolet disinfection guidance manual published by USEPA^101^ provides guidelines on the intensity of UV radiation necessary for the inactivation of pathogenic microorganisms, which vary according to the species or type. For *E. coli* and thermotolerant coliforms, for example, UV radiation of 30–40 mJ cm^–2^ is required, while 50–100 mJ cm^–2^ is recommended for *Giardia*, and up to 250 mJ cm^–2^ for *Cryptosporidium*. Given these values, the dose of UV radiation produced by the lamp used in the present study was more than adequate to neutralize most deleterious pathogens, even considering losses resulting from incrustations, hydraulic inefficiency, and the degradation of the lamp.^101^

This manual also recommends that the UV lamps (in particular, the quartz sleeves) should be cleaned as regularly as demanded by the quality of the water and the observed performance of the lamp. A weekly or fortnightly inspection of the lamp is also recommended for systems without automatic cleaning devices, when the water produced by the system is of reduced quality.^101^

The findings of the present study indicate the need for a complete cleaning of the storage tank and the replacement of the filter cartridge (5 μm) every six months, as a preventive safety measure, considering that the system operated satisfactorily during 10 consecutive months without any interventions. As the UV lamp used in the present study has an estimated lifespan of approximately 8700 h (around one year of continuous operation), annual replacement is recommended to ensure the long-term continuity of the disinfection process.

## 4 Conclusions

The IRGRS (Integrated Rainwater and Greywater Recycling System) evaluated in this study demonstrated significant potential to supply nonpotable uses in a single-family residence. Following the treatment intervention, which included the implementation of UV disinfection and continuous aeration, water quality parameters remained within national and international standards for domestic and agricultural nonpotable reuse. Turbidity maintained daily values below 5 NTU, stabilizing under 0.5 NTU between October 2024 and May 2025. Thermotolerant coliforms were absent, with sporadic occurrences of up to 2 MPN.100 mL^–1^. COD levels were consistently below the minimum detection limit of the method (<5 mg·L^–1^). Surfactants average around 10 mg·L^–1^ weekly, exceeding international limits but remaining compatible with end uses such as floor, vehicle, and laundry cleaning, where their presence is desirable. Complementary parameters (pH, temperature, and electrical conductivity) also remained in compliance with reuse quality guidelines.

Average potable water savings of 41% (minimum 28% and maximum 51%) were recorded throughout the evaluation period. In terms of supply effectiveness, the system achieved satisfactory performance, and even during a six-month period without rainfall, greywater alone was sufficient to meet the household demand (average of 4.1 m^3^·month^–1^). This demonstrates that the system fulfilled its primary objective of ensuring self-sufficiency for nonpotable supply during critical drought periods, when the water bodies used for public and industrial supply in the city reached critical scarcity levels. During the rainy season, underutilization of stormwater was observed, indicating potential for additional uses of the overflow volume.

This study contributes to the advancement of literature by challenging the widely accepted recommendation of daily greywater disposal to prevent bacterial growth during storage. The findings demonstrate that, when subjected to appropriate treatment, greywater can maintain satisfactory microbiological quality, thereby expand its potential applications and reinforce the role of IRGRS as a sustainable alternative for water resource conservation. Furthermore, integrating rainwater and greywater storage in a single reservoir reduces the system’s footprint and prevents the tank from remaining empty and idle during the dry season, thus avoiding infrastructure deterioration. This design feature is particularly advantageous in regions with well-defined seasonal climates, characterized by alternating rainy summers and dry winters (or vice versa).

## Supplementary Material



## Notes

Results of the Student’s *t*-test for recycled water quality data throughout the entire monitoring period; guidelines for the physicochemical and microbiological parameters of recycled water destined for agriculture and urban use; illustrative figures of the system (PDF)
