# Supplementary material for: Long-Term Evaluation of Water Quality and Quantity in a Residential Integrated Rainwater and Greywater Recycling System with Simultaneous Storage and Treatment
Source: ACS Omega. 2026 Mar 2;11(10):16531–45. doi: 10.1021/acsomega.5c12823 (PMC13000656; doi:10.1021/acsomega.5c12823)
Supplement: Supplementary file 1 [file ao5c12823_si_001.pdf]

Supporting Information

Long-term evaluation of water quality and quantity in a residential integrated rainwater and greywater recycling system with simultaneous storage and treatment

Andriane de Melo Rodrigues[a,b], Edio Damásio da Silva Júnior[b*] and Klebber Teodomiro Martins Formiga[a]

[a]Post-Graduate Environmental Science Program, Federal University of Goiás, Esperança Avenue, Goiânia 74690-900, Brazil
[b]Goiano Federal Institute, 88, 310, Setor Sul, P.O. Box 50, Goiânia, Goiás, 74.085-010, Brazil
*Corresponding author. E-mail: edio.damasio@ifgoiano.edu.br

Table S1. Results of the Student's t-test for recycled water quality data throughout the entire monitoring period, comparing outcomes between the wet and dry seasons, both before and after the implementation of ultraviolet disinfection and continuous aeration.

**Parameter**	**Season**	**Before UV&Aeration Mean (n)**	**After UV&Aeration Mean (n)**	**t-value**	**p-value**
Turbidity (NTU)	Rainy	2.28 (60)	0.44 (149)	8.01	< 0.001
	Dry	15.63 (98)	3.68 (44)	10.98	< 0.001
Conductivity (µS.cm$^{-1)}$	Rainy	104.13 (24)	123.78 (149)	-2.34	0.021
	Dry	391 (99)	511.08 (44)	-4.02	< 0.001
pH	Rainy	6.53 (59)	6.71 (148)	-3.85	< 0.001
	Dry	7.11 (99)	7.50 (44)	-6.15	< 0.001
Temperature (°C)	Rainy	29.19 (59)	29.44 (148)	-0.62	0.480
	Dry	26.70 (98)	30.00 (44)	-6.39	< 0.001
COD (mg.L^{-1})	Rainy	13.78 (8)	5.00 (28)	6.1	< 0.001
	Dry	40.4 (17)	12.00 (9)	3.9	<0.005
Surfactants (mg.L-1)	Rainy	ND	10.07 (28)	ND	ND
	Dry	13.39 (11)	9.67 (8)	4.68	< 0.001
Th. Coliforms (MPN.100 mL^{-1})	Rainy	243.50 (11)	0.68 (28)	1.74	0.007
	Dry	37,381.2 (17)	5.66 (9)	4.05	< 0.001

Table S2. Guidelines for the physicochemical and microbiological parameters of recycled water destined for agriculture and urban use, as defined by selected public authorities in Brazil and other countries, worldwide.

Parameter	South African Guidelines	EPA - Water Reuse Guidelines	ISO 16075-2	ABNT NBR 15527	ABNT NBR 16783	Other Guidelines (agricultural water reuse)
Turbidity (NTU)	0–1 no risk; >5 risks begin	2 (avg)–5 (max), more than three states	2 (avg)–5 (max), food crops eaten raw	<2.0; for less restrictive uses <5	<5	British Columbia, Unrestricted: 2
pH	6.0–9.0 ideal range, no health or taste effects	6.5–7.5 Reclaimed Water	–	6.0–8.0	6.0–9.0	AGWR: 6.2–9.8
Temperature (°C)	>30°C promotes microbial growth	>30°C favors microbial growth	–	–	–	–
Odor	1–5 TON; no or slightly perceptible odor	Reclaimed water should be odorless	–	–	–	–
Electrical Conductivity ($\mu S.cm^{-1}$)	–	–	–	–	–	AGWR: 200–2900
Organic Indicators ($mg.L^{-1}$)	DOC < 5, estimated COD < 10	COD < 50 Reclaimed Water	BOD5: 5–10 (food crops eaten raw)	–	BOD5 <20	Israel: COD <100 NS
Thermotolerant Coliforms ($MPN.100mL^{-1}$)	>10 MPN/100 mL (infectious dose)	2.2–23 (food crops); 23–240 (restricted urban reuse)	10–100 (food crops raw); 200–1000 (processed); 1000–10,000 (non-food)	Absent in 100 mL	≤200 *E. coli*	British Columbia, Restricted (weekly) 200; Unrestricted (daily) 2.2
Surfactants ($mg.L^{-1}$)	–	<1 Reclaimed Water	–	–	–	Italy: 0.5; AGWR (anionic): 0.2

Note: NS = Not Specified; DOC = Dissolved Organic Carbon; COD = Chemical Oxygen Demand; BOD_5 = Biochemical Oxygen Demand (5 days).

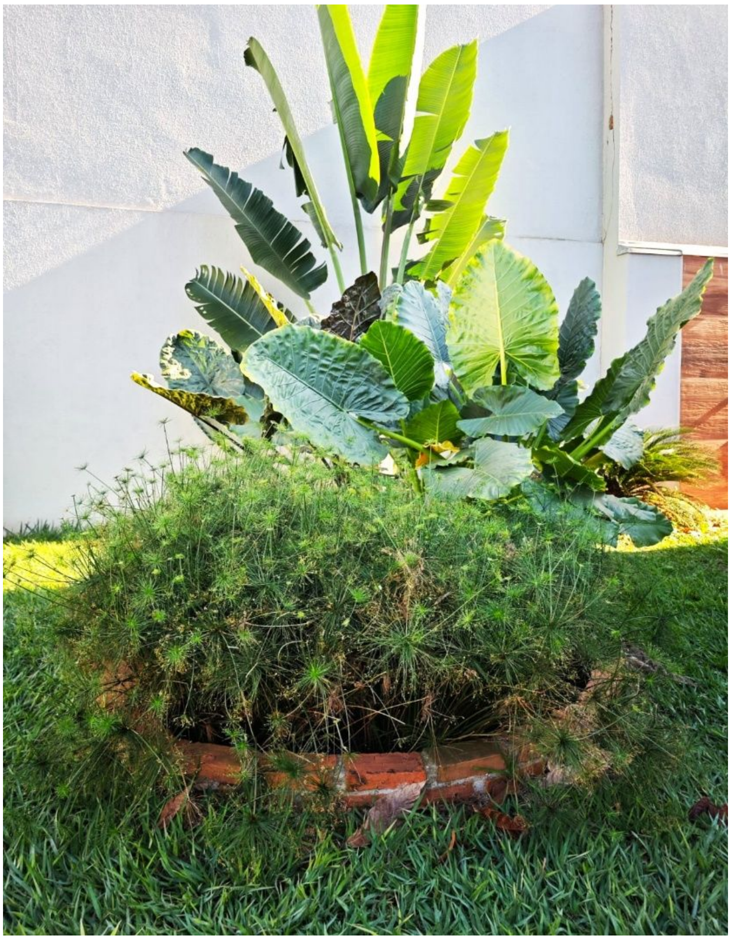

Figure S1. Image captured in May 2025 of the constructed wetland (circular reservoir with exposed brick edges), vegetated with dwarf papyrus.

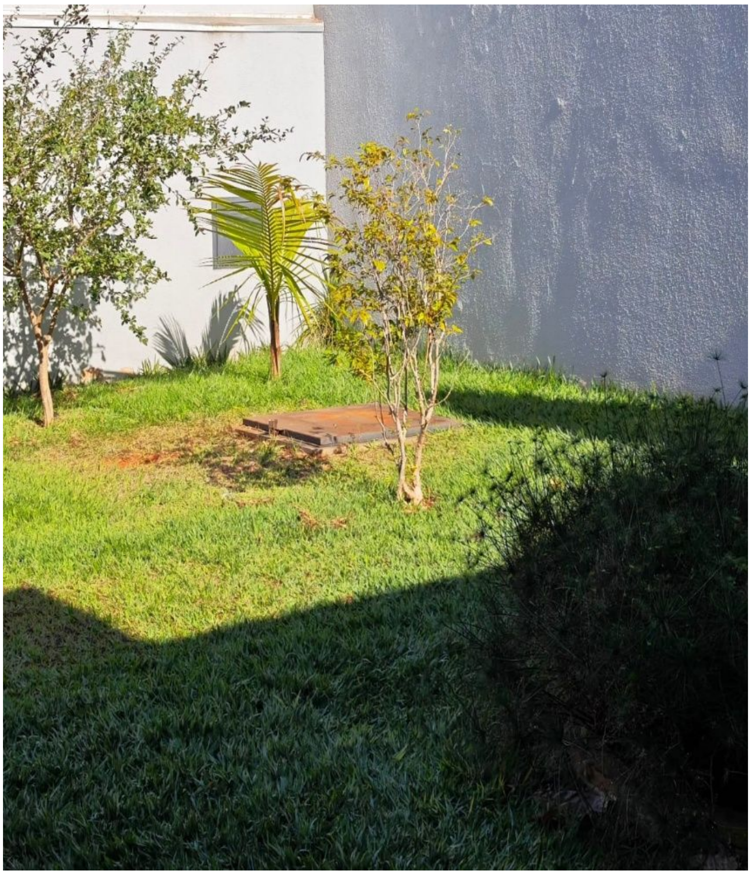

Figure S2. Image captured in May 2025 of the underground reservoir designed for the storage and aeration of rainwater and greywater.

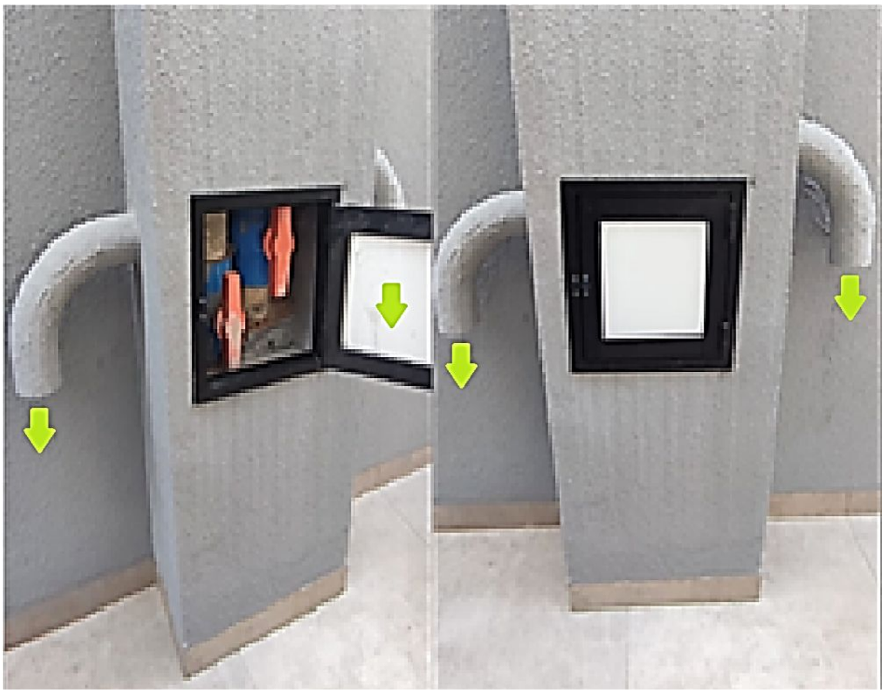

Figure S3. Image captured in June 2025 of the first-flush unit, a system stage used for the pre-treatment of rainwater, shown under manual operation.

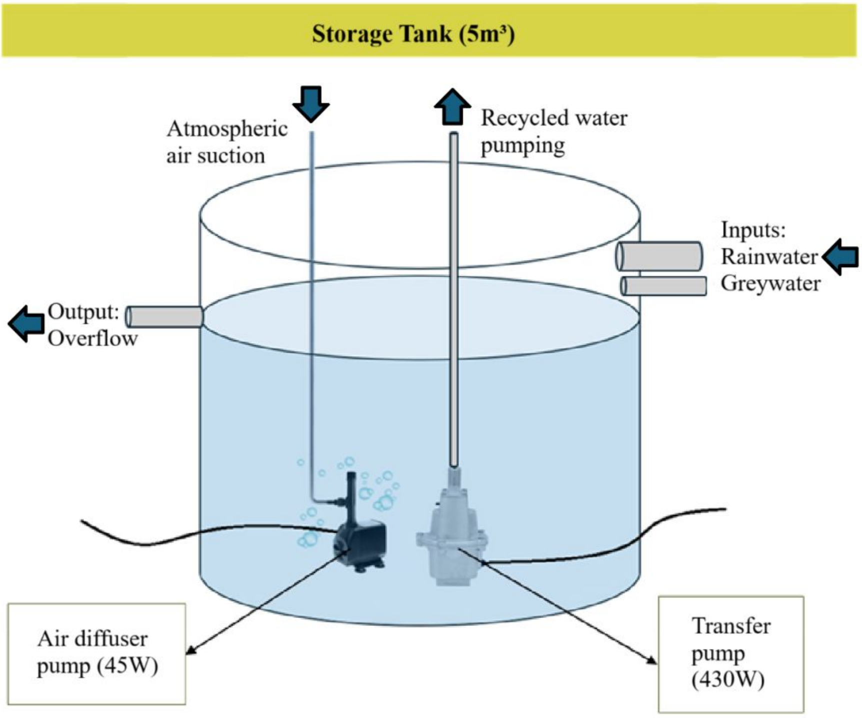

Figure S4. Schematic representation of the storage tank (5 m³), which simultaneously stores rainwater and greywater and treats them through aeration.

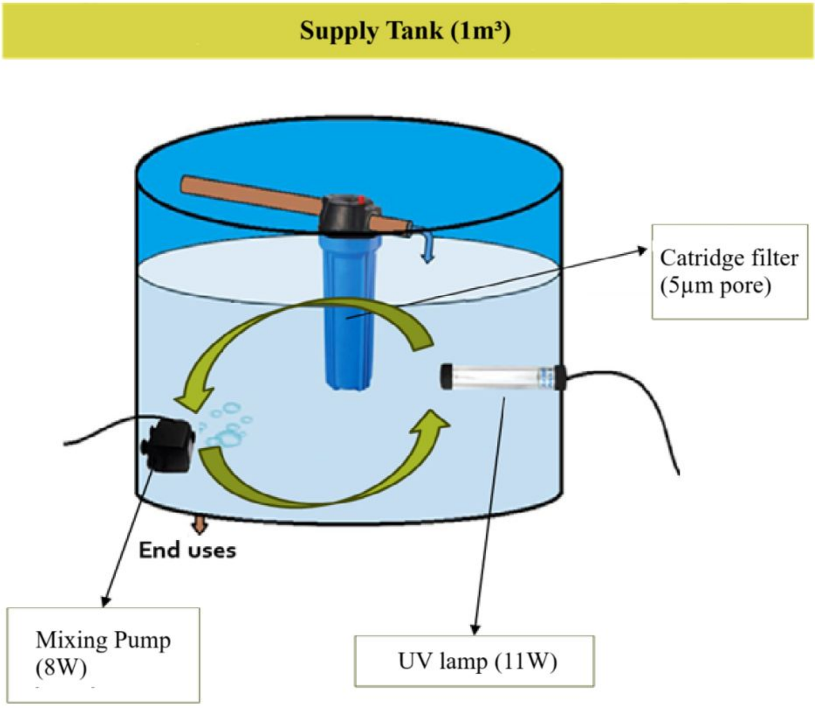

Figure S5. Schematic representation of the supply tank (1 m³), which simultaneously stores and treats rainwater and greywater through filtration (5 µm) and UV disinfection, providing recycled water for end uses including toilet flushing, washing machine, cleaning, and irrigation.
